# A Qualitative Study Exploring the Role of Hindsight Bias in the Process of Reviewing Clinical Practice Prior to Adverse Incidents

**DOI:** 10.1192/bjo.2022.254

**Published:** 2022-06-20

**Authors:** Irene Hadjioannou, Emily Lewis, Leo Scott, Catrin Thomas, Alberto Salmoiraghi, Rajan Nathan

**Affiliations:** 1 Mersey Care NHS Foundation Trust, Liverpool, United Kingdom; 2 Cheshire and Wirral Partnership NHS Foundation Trust, Chester, United Kingdom; 3 Betsi Cadwaladr University Health Board, Ruthin, United Kingdom; 4 Betsi Cadwaladr University Health Board, Wrexham, United Kingdom; 5 University of Liverpool, Liverpool, United Kingdom; 6 Bangor University, Bangor, United Kingdom; 7 University of Chester, Chester, United Kingdom.; 8 Liverpool John Moores University, Liverpool, United Kingdom; 9 National Institute for Health Research, Liverpool, United Kingdom

## Abstract

**Aims:**

To explore the effect of hindsight bias on retrospective reviews of clinical decision making prior to adverse incidents to inform future approaches to incident investigations.

**Methods:**

We have undertaken focus groups with doctors of varying grades across the North West of England and North Wales. A vignette based on a real-life case from the publicly available NHS England Homicide Independent Investigation report database was presented to each group in one of three versions which differed in terms of the ending of the vignettes (i.e. suicide, homicide, no adverse incident). Using a semi-structured interview approach, the group participants were encouraged by the facilitators to reflect on issues relating to risk and risk management. All groups were provided with the same vignette which initially made no reference to the outcome and asked to comment on matters of risk and risk management. Halfway through the discussion, one of the three outcomes was disclosed, and further group discussion was held. The recorded interviews were transcribed and thematic analysis was undertaken using an adapted Framework Method.

**Results:**

Preliminary results (n = 10) indicate that participants identified the potential for significant harm, particularly to others, and identified evidence of key psychopathological and historical correlates to support assertive management of risk and admission to hospital.

Whilst knowledge of the outcome did not lead to participants changing their favoured management plans, it did alter how they appraised the case and led to participants constructing “narrative” explanations for the outcome given. The level of conviction participants held for their management plan reduced when their expectations about the outcome were confounded.

Participants presented with the suicide outcome vignette described their difficulties appraising risk to others and their over-sensitivity to that risk. Participants faced with the ‘no adverse outcome’ vignette perceived the original management plan far more favourably in hindsight. The groups that were presented with the homicide outcome vignette initially focused on both risks to self and others as well as the perceived need for further information. Following knowledge of the outcome, there was a tendency to highlight parts of the letter pertaining to risk to others which they previously had not given as much attention.

**Conclusion:**

The initial analysis of our data confirms the findings from previous studies that hindsight colours the appraisal of adverse events. However, this study is novel in that it describes the nature of the thought processes underpinning the influence of hindsight on appraisals of risk.

